# Profile of stroke patients treated at a rehabilitation centre in Bangladesh

**DOI:** 10.1186/s13104-017-2844-x

**Published:** 2017-10-27

**Authors:** Firoz Ahmed Mamin, Muhammad Shahidul Islam, Farjana Sharmin Rumana, Farhana Faruqui

**Affiliations:** 1 Department of Physiotherapy, Bangladesh Health Professions Institute (BHPI), CRP. Savar, Dhaka, 1343 Bangladesh; 2 Department of Physiotherapy, Centre for the Rehabilitation of the Paralysed (CRP), CRP, Savar, Dhaka, 1343 Bangladesh

**Keywords:** Stroke, Rehabilitation, Bangladesh, Caregivers

## Abstract

**Objective:**

Stroke is the leading cause of death and disability in Bangladesh. Rehabilitation services have not yet been integrated into the Bangladesh health system. Only a few non-governmental organisations provide rehabilitation for stroke patients. The demographic profile of these patients has not yet been established. The aim of this study was to identify and evaluate the socio-demographic data, risk factors, place of primary management and cost of stroke for those who attended rehabilitation at the Centre for the Rehabilitation of the Paralysed (CRP), Bangladesh. A cross-sectional survey was carried out among 103 conveniently selected stroke patients who attended CRP between December 2015 and May 2016.

**Results:**

The mean age of the participants was 49 years. The majority (68%) originated from urban areas. About 85% of the patients had a history of hypertension prior to their stroke. Following the stroke, most patients received their initial treatment in a general clinic or hospital by registered physicians. Only 22% of the patients were advised to pursue follow-up rehabilitation services by their physicians. All patients interviewed in the survey received unpaid full-time care from their family members. The reported cost of rehabilitation was approximately US $328 per month per patient.

**Electronic supplementary material:**

The online version of this article (10.1186/s13104-017-2844-x) contains supplementary material, which is available to authorized users.

## Introduction

The global burden of disease has shifted in the last few decades from infectious and nutritional disorders to non-communicable disease [1]. The incidence of stroke has been increasing throughout the world and is particularly prevalent in developing countries where it is now the second leading cause of death [2]. The numbers relating to stroke related deaths and disability are extremely high in developing countries. Globally, developing countries account for approximately 75% of stroke related deaths and 81% of stroke related disability-adjusted life-years (DALYs) [3]. Bangladesh is a densely populated developing country and faces the double burden of both communicable and non-communicable diseases [4]. Rapid urbanisation, changing dietary habits, a lack of physical activity, consumption of tobacco and a decline in communicable diseases have all contributed to a rising prevalence of chronic diseases such as stroke [5]. Stroke creates a significant burden in an economic and social perspective, and this burden is increasing [6]. Patients often require individualised long-term care and rehabilitation services [7]. Although facilities for acute stroke care have been developing in Bangladesh, rehabilitation services are lagging far behind primary care services [9]. CRP is one of the few centres available for stroke rehabilitation. It should be noted that the demographical information regarding CRP patients is limited [8].

## Main text

### Methods

#### Aim of this study

The aim of this study was to provide an overall view of stroke patients who took-up rehabilitation services at CRP.

The specific objectives were to;

1. Observe the sociodemographic characteristics of attendees.
2. Identify the risk factors to stroke of this group and assess the association between various socio-demographic characteristics.
3. Identify the prior knowledge on stroke, initial management of stroke, information regarding care-givers and the extent of financial cost for stroke patients attending CRP.

A cross-sectional study of stroke patients who attended CRP for rehabilitation services was conducted between December 2015 and May 2016. We selected participants who had a confirmed diagnosis of stroke by a registered physician, who had been utilising rehabilitation services for at least 1 month at CRP and who had agreed to participate in this study. There was no age limit, and both male and female participants were enlisted. Patients who had a history of stroke, transient ischemic attack (TIA) or surgery for stroke prior to their current admission were excluded. A questionnaire was developed and a pilot study was carried out to finalise the questionnaire, with five physiotherapists trained as data collectors. Data was collected from the participants and, where necessary, from their carers through face-to-face interviews. In terms of occupation, those who were employed, self-employed or farmers were categorised as employed, and those who were unemployed, housewives or retired were categorised as unemployed.

We used the descriptive statistics to present the data and Pearson’s Chi square test to identify the differences among various socio-demographic features and risk factors. A Multivariate logistic regression was performed to determine the association between various socio-demographic data and risk factors. Differences and associations were considered significant when p < 0.05. Data analysis was performed using the Statistical Package for the Social Science (SPSS), Version 19.0. Armonk, NY: IBM Corp.

### Results

In the period December 2015 to May 2016, a total of 103 participants were selected for this study based on the selection criteria. Of these individuals, 77.7% were males and the average age was 49 (SD ± 8) years. Detailed socio-demographic information is given in Table 1.

**Table 1:** Socio-demographic features of stroke patients

Variables	Categories	Frequency (N = 103)	Percentage (%)
Gender	Male	80	77.7
	Female	23	22.3
Age (years)	30–40	8	7.8
	41–50	64	62.1
	51–60	21	20.4
	61–70	8	7.8
	71 <	2	1.9
Marital status	Married	99	96.1
	Single	4	3.9
Education	No formal education	6	5.8
	Primary	17	16.5
	Secondary	18	17.5
	Higher secondary	6	5.8
	Third level education and above	56	54.4
Residence	Village	8	7.8
	Suburban	23	22.3
	Urban	70	68.0
Occupation	Employed	68	66
	Self employed	7	6.8
	Farmer	4	3.9
	Unemployed	4	3.9
	Housewife	17	16.5
	Retired	3	2.9

#### Risk factors for stroke

Based on self-reported information, 85% of the study group had high blood pressure, 69% had high blood cholesterol and 77% had diabetes prior to their stroke. About 80% of the patients added salt to their daily meals and almost all of the males (81%) were smokers. Differences and association of various socio-demographic information and risk factors are presented in Tables 2 and 3 respectively.

**Table 2:** Chi square analysis of response variables by selected socio-demographic factors

Variables	High blood pressure χ^2^ (%)	High blood cholesterol χ^2^ (%)	Diabetes χ^2^ (%)	Added salt χ^2^ (%)	Smoking χ^2^ (%)
Gender	χ^2^ = 3.16	χ^2^ = 3.88*	χ^2^ = 1.74	χ^2^ = .59	χ^2^ = 41.36*
Male	88.8	73.8	73.8	81.3	81.3
Female	73.9	52.2	87.0	73.9	0
Age	χ^2^ = 11.17*	χ^2^ = 12.38*	χ^2^ = 4.41*	χ^2^ = .63	χ^2^ = 9.88*
Below 60	89.2	74.2	79.6	80.6	69.9
Above 60	50.0	20	50	70	20
Education	χ^2^ = 11.91*	χ^2^ = 28.08*	χ^2^ = 10.87*	χ^2^ = 17.08*	χ^2^ = 47.27*
Below third level education	72.3	42.6	61.7	61.7	29.8
Above third level education	96.4	91.1	89.3	94.6	94.6
Occupation	χ^2^ = 8.86*	χ^2^ = 10.86	χ^2^ = .60	χ^2^ = 8.72*	χ^2^ = 58.23*
Employed	91.1	77.2	78.5	86.1	84.8
Unemployed	66.7	41.7	70.8	58.3	.0
Residence	χ^2^ = 8.80*	χ^2^ = 27.07*	χ^2^ = 22.94*	χ^2^ = 1.28	χ^2^ = 9.94*
Rural and semi urban	69.0	31.1	44.8	72.4	41.4
Urban	91.19	83.8	89.2	82.4	47.3

* p < 0.05

**Table 3:** Binary logistic regression presenting the relationship of selected socio-demographic factors on various risk factors of stroke

Variables	Hypertension AOR (95% CI); p	High blood cholesterol AOR (95% CI); p	Diabetes AOR (95% CI); p	Added salt AOR (95% CI); p	Smoking AOR (95% CI); p
Gender					
Male	2.78 (.87–8.88); .08	2.57 (.98–6.71); .053	.42 (.11–1.56); .19	1.52 (.51–4.53); .444	45.50 (9.60–215.50); .000*
Female					
Age					
Below 60 years	8.30 (2.04–33.74); .003*	11.50 (2.28–57.97); .003*	3.89 (1.02–14.84); .046*	1.78 (.42–7.59); .43	9.28 (1.85–46.52); .007*
Above 60 years					
Education					
Below third level education	.09 (.021–.45); .003*	.07 (.02–.21); .000*	.19 (.06–.54); .002*	.09 (.02–.33); .000*	.024 (.00–.09); .000*
Above third level education					
Occupation					
Employed	5.14 (1.62–16.24); .005*	4.74 (1.80–12.47); .002*	1.50 (.53–4.21); .439	6.81 (2.35–19.78); .000*	35.65 (10.09–92.47); .000*
Unemployed					
Residence					
Rural and semi urban	.19 (.06–.61); .005*	.087 (.032–.237); .000*	.09 (.03–.27); .000*	.55 (.20–1.53); .260	.24 (.09–.60); .002*
Urban					

*OR* adjusted odds ratio, *CI* confidence interval

* p < 0.05

This data shows that the risk factors for stroke were consistently higher and significant amongst the younger, urban dwelling, well-educated and employed participants. Males had a statistically greater prevalence for high cholesterol and smoking than females. However it should be noted that, culturally, women do not smoke in Bangladesh.

#### Previous knowledge on stroke

About 73% of the patients reported that they knew they were at risk of stroke. 61% of the patients had been advised by a physician that their comorbidities put them at risk of stroke. However, only 7% of participants attended their doctors regularly for their health conditions, and 93% patients did not see doctor until they felt unwell. This study suggests that the education level of participants positively correlated with a prior knowledge of stroke, as presented in Additional file 1.

#### Initial management of stroke of those attending CRP

About 90% of the patients received immediate care in a general hospital or clinic upon onset of their stroke and the remaining 10% were treated at home by untrained traditional medical practitioners. On presentation for those attending hospital or clinic, 96% underwent diagnostic imaging, either via Magnetic Resonance Imaging (MRI) or Computerized Tomography (CT). About 92% of patients discussed their results with a neurologist. After their incident, about 84% of patients were initially managed by registered physicians, with 10% seen by unqualified traditional village doctors. About 50% of patients received their stroke treatment at tertiary level hospitals. Of the study group, only 22% had been advised to seek further rehabilitation at CRP by their physician. 61% of the group was recommended to attend CRP by previous patients and 11% by acquaintances.

#### Information regarding care-givers

All the patients had personal care-givers. 68% of male patients were cared for by their spouses, the majority being housewives. For female patients, daughters accounted for 80% of care-givers in this demographic.

#### Financial cost of stroke rehabilitation

Stroke is an extra financial cost for a family; the participants in this study reported that their average monthly expenditure was US $328 for rehabilitation services alone. Only 10% of patients received any sort of financial aid, usually from their employers, with the remainder paying independently.

## Discussion

In this study, we provided a snapshot demographic view of patients attending CRP for stroke rehabilitation. In addition to recording age, gender and area, we also attempted to identify those who had comorbidities that could contribute to the risk of stroke, and ascertain whether the patients had any knowledge of stroke prior to its occurrence.

The socio-demographic information revealed that the group most at risk to stroke are males under the age of 60 years, living in urban areas and educated to a third level.

Though most commonly associated with the elderly [9], the global incidence of stroke affecting those below the age of 45 years is significantly higher in low income, developing countries. In these countries, up to 30% of all strokes occur in people under 45, compared to only 5% in western countries [10]. The average age of the participants in our study was 49 years, with 69.9% below the age of 50. This figure is low in comparison to the mean age of stroke patients in many other countries, including neighbouring India [9, 11].

Many participants in this study had stroke risk factors prior to onset. Mohammad et al. data on stroke patients from different hospitals in Bangladesh showed 57.6% of patients had a history of hypertension, 23% had diabetes, 44.6% had a history of smoking [12]. Our results for these risk factors were greater, however, at 85%, 77% and 81% respectively. Further investigation is required to determine the causes behind this trend. We found that there was a significantly higher prevalence of hypertension and diabetes among higher-educated employed people aged between 41 and 60. Among the participants as a whole, about 70% knew that they were at risk from stroke, and 61% of the group had had these risks highlighted to them by a physician. However, only 7% of the patients visited a doctor prior to their stroke. Amongst those who had received higher education, the majority considered themselves well informed about stroke, and most (92%) had had their risk factors highlighted to them by a physician. Thus, it can be inferred that the lifestyle and health behaviour of educated people are not conducive to healthy lifestyle choices. It appears that participants who had been informed of the risk of stroke failed to take any preventative measures, and this has been found to be common amongst patients with chronic disorders [13].

Despite the fact that only 34.27% [14] of the population of Bangladesh live in urban areas, 68% of the participants of this study lived in urbanised areas. These findings support the study by Hossain et al. which found that most patients treated for stroke in Bangladeshi tertiary level hospitals came from urban areas [15]. Fatema et al. suggests that the prevalence of cardiovascular disease in urban areas is a potential reason for an increase in this demographic [16]. It can be assumed that educated individuals, living in urban areas and with an occupation which is less physically active, adopt additional poor lifestyle choices such as smoking. We conducted this study at CRP, which is near Dhaka, the capital of Bangladesh, which may account for the prevalence of urban-dwelling, educated, employed participants. We would need a further countrywide study to determine the correlation between stroke and urban dwelling.

This study found that the majority of participants received their initial care at a hospital or a clinic and were treated by a registered physician. Despite the scarcity of trained neurologists and neuro-imaging facilities in Bangladesh, 92% of patients in this study reported that they had received treatment by neurologists and nearly all patients had undergone either an MRI or CT scan for their condition.

This study also found that most patients who attended CRP had been recommended to do so by patients who were beneficiaries of CRP’s services previously. Only 22% of patients had been advised to seek rehabilitation by their physician. In our study, all patients received full care from their family members, either from a spouse or daughter. Dewey et al. found that approximately 74% of stroke survivors cannot manage their daily activities without assistance and, typically, family members were required to provide this care [17]. According to this study, wives and daughters were the main carers for men and women respectively and found that no patients had utilised the services of paid professional caregivers. In terms of occupations, the wives were predominantly housewives. Lutz et al. states that caring for stroke patients is a difficult task and has a huge impact on a caregiver’s own health and wellbeing [18].

There is also a financial cost associated with providing care for a family member with a stroke. Heeley et al. states that stroke creates considerable economic hardship, particularly when the individuals had been the main financial earners of the family [19].By the time they arrived at CRP, patients had already paid considerably for their stroke management. In addition to their typical monthly expenditure families, had to pay an average US $328 per month to support their rehabilitation while at CRP. At the time of the study, the average annual income in Bangladesh was US $1466 [20]. Thus, the average monthly expenditure for a participant’s rehabilitation equates to more than one-fifth of their annual income. About 90% patients were dependent on their families to bear this expense and did not receive any organisational or governmental help. Previous studies have found that these additional costs exacerbate the poverty of people in many developing countries, such as Bangladesh [21]. Though the financial impact of health care cost depends on the financial capacity of people, it is estimated that annually about 4.2% of people are forced into extreme poverty to meet healthcare expenditure in Bangladesh [22].

## Limitations

This study consisted of a small cohort of participants, and focused on a single rehabilitation facility. We assumed that the participants’ accounts of their healthcare journey and healthcare conditions were accurate. We provided a very broad overview of a range of demographics and their links to the participants’ health conditions. An in-depth study could be of great benefit.

## Additional file

**Additional file 1: Table S1.** Level of education and awareness of the presence of stroke risk factors.

## Abbreviations

DALYs: disability-adjusted life years
CRP: Centre for the Rehabilitation of the Paralysed
TIA: transient ischemic attack
IRB: Institutional Review Board
BHPI: Bangladesh Health Professions Institute
SPSS: Statistical Package for the Social Sciences
MRI: magnetic resonance imaging
CT scan: computed tomography scan
OR: odds ratio
CI: confidence interval
