# Supplementary material for: Profile of stroke patients treated at a rehabilitation centre in Bangladesh
Source: BMC Res Notes. 2017 Oct 27;10:520. doi: 10.1186/s13104-017-2844-x (PMC5658960; doi:10.1186/s13104-017-2844-x)
Supplement: Supplementary file 1 — Additional file 1: Table S1. Level of education and awareness of the presence of stroke risk factors. [file 13104_2017_2844_MOESM1_ESM.docx]

**Table S 1: Level of education and awareness of the presence of stroke risk factors**

| **Level of Education** | **Percentage (%)** | **χ^2^** | **p** | **AOR** | **CI (95%)** | **p** |
| --- | --- | --- | --- | --- | --- | --- |
| Below Third level education | 42.6 | 33.58* | .000* | .042 | (.011-.154) | .000* |
| Above Third level education | 94.6 |  |  |  |  |  |

*p<0.05, AOR: Adjusted Odds Ratio, CI: Confidence Interval
